# Optimization of diagnostic imaging use in patients with acute abdominal pain (OPTIMA): Design and rationale

**DOI:** 10.1186/1471-227X-7-9

**Published:** 2007-08-06

**Authors:** Wytze Laméris, Adrienne van Randen, Marcel GW Dijkgraaf, Patrick MM Bossuyt, Jaap Stoker, Marja A Boermeester

**Affiliations:** 1 Department of Surgery, Academic Medical Center, University of Amsterdam, The Netherlands; 2 Department of Radiology, Academic Medical Center, University of Amsterdam, The Netherlands; 3 Department of Clinical Epidemiology Biostatistics, and Bioinformatics, Academic Medical Center, University of Amsterdam, The Netherlands

## Abstract

**Background:**

The acute abdomen is a frequent entity at the Emergency Department (ED), which usually needs rapid and accurate diagnostic work-up. Diagnostic work-up with imaging can consist of plain X-ray, ultrasonography (US), computed tomography (CT) and even diagnostic laparoscopy. However, no evidence-based guidelines exist in current literature. The actual diagnostic work-up of a patient with acute abdominal pain presenting to the ED varies greatly between hospitals and physicians. The OPTIMA study was designed to provide the evidence base for constructing an optimal diagnostic imaging guideline for patients with acute abdominal pain at the ED.

**Methods/design:**

Thousand consecutive patients with abdominal pain > 2 hours and < 5 days will be enrolled in this multicentre trial. After clinical history, physical and laboratory examination all patients will undergo a diagnostic imaging protocol, consisting of plain X-ray (upright chest and supine abdomen), US and CT. The reference standard will be a post hoc assignment of the final diagnosis by an expert panel. The focus of the analysis will be on the added value of the imaging modalities over history and clinical examination, relative to the incremental costs.

**Discussion:**

This study aims to provide the evidence base for the development of a diagnostic algorithm that can act as a guideline for ED physicians to evaluate patients with acute abdominal pain.

## Background

Acute abdomen is a common presentation in emergency medicine. It represents 5 to 10% of all Emergency Department (ED) visits [1,2]. Causes of acute abdominal pain can range from benign self-limiting conditions to acute life-threatening disorders. To provide appropriate triage and care, rapid and accurate diagnostic work-up is necessary.

No sufficient data are currently available in the literature to develop evidence-based guidelines for diagnostic imaging in patients with acute abdominal pain at the ED. Current diagnostic work-up shows wide variation in the use of imaging modalities, both between and within hospitals. Plain X-rays, ultrasonography and computed tomography are used, if used at all, solitary or combined in patient work-up. During the last decade, a trend towards increased use of computed tomography in patients with abdominal pain [3-5] can be seen. New questions concerning cost-effectiveness of imaging have been raised, with regards to both over-usage and under-usage of imaging.

Over-usage may have a negative impact on efficient patient throughput, costs and radiation exposure. Under-use may delay treatment, which in urgent conditions can increase morbidity and even mortality. Some physicians advocate diagnostic laparoscopy instead of imaging in patient work-up. Diagnostic laparoscopy has a high accuracy in highly selective patient series. Because of its invasiveness, costs and complications [6] laparoscopy is not suitable as an initial diagnostic tool in patients with acute abdominal pain at the ED when similar accuracy can be obtained without surgery.

Most research performed so far focused on diagnostic value of imaging for specific diagnosis. Imaging in acute appendicitis is a topic well-studied, but represents only 6 to 10% of ED presentations of abdominal pain [2,7]. The purpose of this study (OPTIMA trial) is to collect data for constructing an optimal diagnostic algorithm for the wide spectrum of patients with acute abdominal pain at the ED. Using this algorithm, decisions to perform further imaging can be based on patient characteristics, signs and symptoms at presentation without compromising health care by missing relevant diagnosis or delaying treatment. Optimizing usage of imaging modalities will reduce costs and may allow the development of an evidence-based guideline for patients presenting with acute abdominal pain at the Emergency Department.

## Methods/design

### Study objectives

The study aims to develop optimal diagnostic algorithms for the acute abdomen, based on the added value of plain x-ray, US and CT over clinical history, physical and laboratory examination. Furthermore, we will perform a cost-effectiveness analysis of imaging use.

### Study design

OPTIMA is a fully paired diagnostic accuracy study, with a complete diagnostic protocol for patients with acute abdominal pain. All included patients will undergo an abdominal and chest X-ray, abdominal ultrasound and a CT abdomen with intravenous contrast medium. The reference standard is the final diagnosis assigned by an expert panel with all individual information available including 6 months of follow-up.

### Setting

Patients will be enrolled from 6 participating Dutch hospitals, including two university hospitals (Academic Medical Center, AMC and University Medical Center Utrecht, UMCU) and four large teaching hospitals (Antonius Hospital Nieuwegein (AZN), Onze Lieve Vrouwe Gasthuis (OLVG), Gelre hospital and Tergooi hospitals).

### Study group

A random selection of consecutive adult patients who are presented, by themselves or a general practitioner, to the emergency department with non-traumatic abdominal pain persisting for at least 2 hours and less than 5 days.

### Eligibility criteria

#### Inclusion criteria

- Patients with abdominal pain with a duration of > 2 hours and < 5 days presenting at the ED.

#### Exclusion criteria

- Age < 18 years.

- Pregnancy.

- Abdominal pain due to blunt or penetrating trauma.

- Hemorrhagic shock caused by gastrointestinal bleeding or ruptured aortic aneurysm.

- Patients in whom no imaging was warranted by the treating physician ánd who were subsequently discharged home from the ED.

### Ethics and informed consent

The independent medical ethics committee (MEC) of the initiating hospital approved our final protocol, after consulting the advisory board on radiation exposure. The radiation dose, related mostly to the CT-scan, is within the guidelines of the MEC. An abdominal CT scan, with an estimated effective dose of 10 mSv, raises the possibility of X-ray induced fatal cancer by 0.05%, in addition to a baseline life time risk of naturally induced fatal cancer of 20% in the US [8].

All five other participating hospitals gave their approval after assessing the local feasibility of this study. Written informed consent will be obtained from all patients.

### Diagnostic tests

Initial examination will consist of standardized clinical history, physical and laboratory examination. Subsequently, a supine abdominal X-ray, an upright chest X-ray, an abdominal ultrasound and a spiral CT scan will be performed.

The diagnostic tests are also performed in a standardized way. Abdominal ultrasound scanning will systematically investigate the entire abdomen for general and organ specific anomalies. Both positive and negative findings will be recorded of all variables listed in the case record form. A curved 3.5 – 5.0 MHz array and a linear 10 MHz array will be used.

All abdominal CT scans will be performed using a multi-detector row 4 or 16 slice helical CT scanner (SOMATOM Sensation 16 and SOMATON plus; Siemens Medical Systems, Forchheim, Germany; MX8000 and Tomoscan AV; Philips Medical Systems, Best, The Netherlands). The model CT scan protocol consists a scan with an effective mAs level of 165, and 120 kV, collimation: 2.5 mm, slice width: 3 mm, rotation time: 0.5s. Intravenous contrast (125 ml. Visipaque 320; Amersham Health AS, Oslo, Norway) will be injected at 3 ml/s. Scanning will start after 60 seconds. No oral or rectal contrast agents will be used.

**Figure 1:**
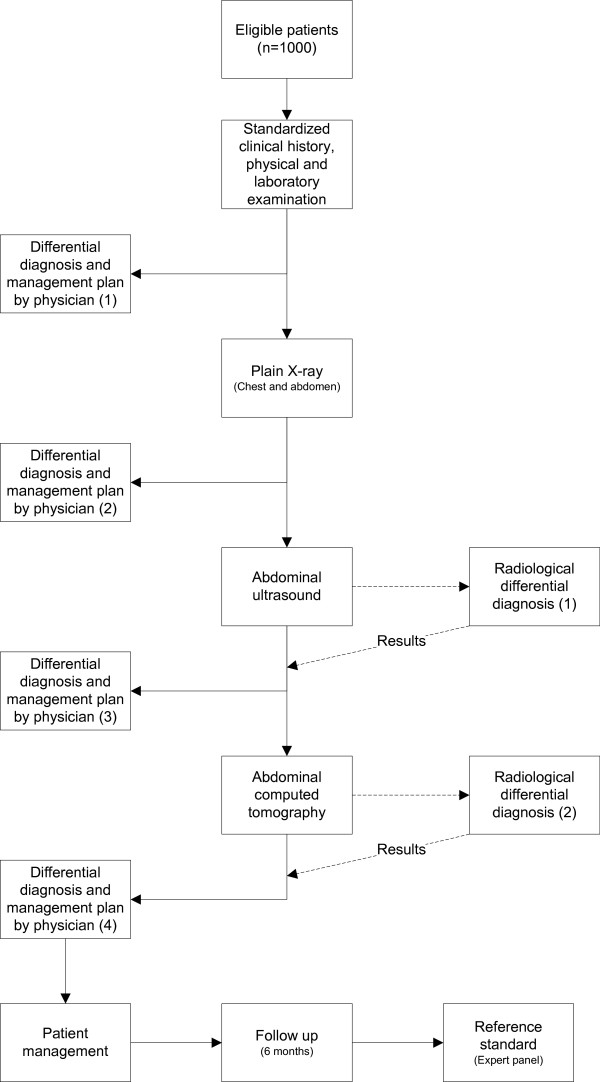
shows the OPTIMA study flow chart.

All results, including findings and diagnosis after initial examination, will be recorded independently of previous results and other findings. Case Record Forms (CRF) will facilitate the standardization of clinical history, physical examination, laboratory parameters and radiological examination. After clinical history, physical and laboratory examination the three most likely diagnoses, a level of confidence of the most likely diagnosis and a management plan will be recorded by the treating physician. Subsequently, a differential diagnoses, level of confidence of the most likely diagnosis and a management plan will be recorded separately after plain X-ray, after US and finally after CT (Figure 1).

Chest and abdominal X-rays will be evaluated by the treating physician. Both US and CT are performed and evaluated by radiological residents or radiologists, blinded for each others test results and for the test results of the abdominal and chest X-rays. Summarized clinical findings, as in routine practice, will be provided to the radiologist. The radiologist performing the ultrasound will record the findings in the patients CRF with general and organ-specific US findings ending with a differential diagnosis with a level of confidence of the most likely diagnosis. Another radiologist evaluates the CT scan and records data in a similar way.

### Outcome measures

#### Follow up

Six months of follow up will be obtained for all patients. All available information will be gathered, including course of disease, laboratory findings, operation reports, pathology reports, treatment outcome and costs.

#### Reference standard

All included patients will be evaluated using a uniform reference standard not dependent of the index tests results. An expert panel will review each case and assign the final diagnosis. Cases will be presented in a standardized way, including all available follow-up data gathered as mentioned above. Panel members will evaluate cases individually, after which consensus is reached in group discussion. These final diagnoses will be classified as urgent, defined as a diagnosis which requires treatment within 24 hours, or as non-urgent. The distribution of conditions based on urgency is presented in table 1.

**Table 1:** Urgency classification of diagnoses

**Urgent**	**Non-urgent**
Acute appendicitis	Cholecystolithiasis
Acute diverticulitis	Obstipation
Perforated vicus	Gastrointestinal disorders^∥^
Bowel obstruction/ileus	Hepatic disorders^¶^
Bowel ischemia	Malignancy**
Inflammatory bowel disease	Gynaecological disorders^††^
Peritonitis	Renal and urinary tract disorders^‡‡^
Acute cholecystitis	Non-specific abdominal pain
Cholangitis	
Pancreatitis (acute and chronic)	
Abscess*	
Renal and urinary tract disorders^†^	
Gynaecological disorders^‡^	
Extra-abdominal disorders^§^	

* Intra-abdominal abscess, retro-peritoneal abscess, hepatic abscess, tubo-ovarian abscess

^† ^Renal and ureteral stones with obstruction, hydronephrosis, pyelonephritis

^‡ ^Adnexal torsion, pelvic inflammatory disease, bleeding/rupture ovarian cyst

^§ ^Pneumonia, myocardial infarction, mediastinitis

^∥ ^Gastritis, gastroenteritis, peptic ulcer, acute epiploic appendagitis

^¶ ^Hepatitis, hepatic metastases

** Pancreatic, intestine and kidney malignancies

^†† ^Ovulation pain/bleeding, endometriosis, menstrual pain, uterine myoma

^‡‡ ^Renal and ureteral stones without obstruction, urinary tract infection

### Data analysis

In the analysis, focus will be on the added value of plain X-ray, US and CT over clinical history, physical examination and laboratory findings, in the timely identification of urgent conditions. For this purpose, the findings from the respective imaging techniques will be cross-classified with the urgency categorization. To make head-to-head comparisons between modalities, sensitivities, specificities, likelihood ratios and predictive values will first be calculated for each imaging technique separately.

Next, we will evaluate the diagnostic performance of more detailed diagnostic algorithms. These diagnostic algorithms will be developed using logistic regression modelling. Additional models will look at the incremental gains in the detection of urgent conditions or at the rightful refutation of suspected urgent conditions through imaging modalities and combinations of imaging modalities. A guiding principle is that, in case of equality in diagnostic performance, the diagnostic strategy associated with fewer costs and lower patient burden is to be preferred.

Efficacy of imaging strategies will be investigated for specific patient profiles based on for example age, gender, localisation of pain and organ failure found at physical and/or laboratory examination. A diagnostic strategy may therefore alter for different patient profiles.

Subgroup analysis for specific, frequently occurring, diagnosis will be performed. For example, sub-group analysis for patients diagnosed with appendicitis or diverticulitis. Such analyses will use logistic regression modelling to identify predictive diagnostic variables in the clinical history, physical examination and laboratory findings. As we expect to include a substantial number of patients with non-specific abdominal pain, another analysis will be focussed on the early identification of this subgroup. Herewith also logistic regression modelling will be used to identify discriminate factors in history, physical and laboratory examination and imaging findings.

### Sample size

This study is powered to determine sensitivities of the respective imaging modalities with the 95% confidence level not extending the anticipated base sensitivity of 80% with more than 5%. We calculated that 246 patients with an urgent condition will be needed. Given the anticipated 25% urgent cases, at least 1,000 patients are needed in this study. This sample size will also provide sufficient data about rare conditions. Judging by the literature search on acute abdomen performed during preparatory activities prior to this study, we expect to include 110 patients (11%) with less frequent diagnoses. Sample size heuristics for multivariable modelling indicate that including 1,000 patients allows the inclusion of at least 15 variables covering both initial presentation and imaging results.

### Economic evaluation

For the economic evaluation we will obtain in-hospital direct medical costs. Direct and indirect non-medical costs will be collected by two patient questionnaires sent to the participants three and six months after hospital admission.

We will compare the diagnostic accuracy and the costs of several imaging strategies. We anticipate an imaging strategy with less imaging but the same accuracy as the reference strategy (all imaging in all).

## Discussion

### Rationale behind the study design

An alternative, possibly preferable study design is that of a randomised control trial (RCT). However, comparing all possible and daily used diagnostic strategies will need numerous trial arms with large amounts of patients and therefore is not practically feasible. Randomising patients to diagnostic strategies that are not evidence-based exceeds our study objectives. Our study is designed to collect evidence to provide guidelines for such diagnostic strategies. Therefore we chose this design which contains a full, probably over-complete, diagnostic protocol of 1,000 consecutive patients. Comparing imaging modalities separately as well as subsequently is possible because of stepwise and independently recording of the diagnosis, level of confidence of the diagnosis and a management plan after each diagnostic step.

The accurate detection of urgent conditions will lead to better therapeutic decision making. By making this a basic premise in our statistical analysis we believe that the optimal diagnostic strategy will lead to the health gains. However, our design prevents an empirical comparison of the health outcomes of different strategies. Because of our complete diagnostic protocol it does not allow us to document consequences in patient outcome in case of false positive or false negative radiological findings. A RCT would allow us to estimate these outcome consequences, but at the price of a major limitation in the number of comparable strategies.

The reference standard in a diagnostic study is generally a challenge, because patients with a positive index test tend to receive a different reference test (e.g. surgery) than patients with a negative index test (e.g. clinical follow-up). This verification bias can lead to overestimation of the diagnostic test accuracy. Therefore, we chose a uniform reference standard for all patients. By means of an expert panel, in which radiologists and surgeons experienced in acute abdominal conditions will assign the reference diagnosis post hoc.

Diagnostics, both clinical and radiological, will be performed by residents as well as specialists. This reflects daily practice in participating hospitals and will make our results more generalisable. The influence of experience of the investigator on accuracy results will additionally be studied in our analysis. Random selection of consecutive patients diminishes the risk of spectrum and selection bias. We believe that the definition of acute abdomen as used in this study renders the inclusion of cases across the whole clinical spectrum of patients with acute abdominal pain. Results obtained from our study population will therefore be applicable to the general population presenting with acute abdominal pain at the ED.

While a full consecutive series of patients is impractical, a random selection of consecutive patients is made in each participating centre. This selection depends on each hospitals case-load and capacity of imaging resources, but is independent of physician or patient preference. Limited capacity at the Department of Radiology forces some participating hospitals to include a maximum of 2 or 3 patients a day. Physician preference is minimized by daily including the first consecutive presenting eligible patients with acute abdominal pain. Selection bias may be caused by variation in policy to discharge patients presenting with acute abdominal pain from the ED without any further imaging. Such threshold can differ between hospitals. Observer bias will be minimized because of standardized evaluation of imaging independently of preceding test results.

Our study design will provide us the evidence base for constructing optimal diagnostic guidelines for patients with acute abdomen.

## Conclusion

The OPTIMA trial is a fully paired diagnostic accuracy study that aims to develop an optimal diagnostic strategy, based on a cost-effectiveness analysis of imaging use, for patients with acute abdominal pain presenting at the ED. Results are expected early 2008.

## Competing interests

The author(s) declare that they have no competing interests.

## Authors' contributions

WL drafted the manuscript. AVR co-authored the writing of the manuscript. MAB, JS, PMMB and MGWD designed the study and co-authored the writing of the manuscript.

## Pre-publication history

The pre-publication history for this paper can be accessed here:


